# Inhibition of *ICAM2* induces radiosensitisation in oral squamous cell carcinoma cells

**DOI:** 10.1038/sj.bjc.6604290

**Published:** 2008-03-18

**Authors:** T Ishigami, K Uzawa, K Fushimi, K Saito, Y Kato, D Nakashima, M Higo, Y Kouzu, H Bukawa, T Kawata, H Ito, H Tanzawa

**Affiliations:** 1 Department of Clinical Molecular Biology, Graduate School of Medicine, Chiba University, 1-8-1 Inohana, Chuo-ku, Chiba 260-8670, Japan; 2 Division of Dentistry and Oral-Maxillofacial Surgery, Chiba University Hospital, 1-8-1 Inohana, Chuo-ku, Chiba 260-8670, Japan; 3 Department of Radiology, Graduate School of Medicine, Chiba University, 1-8-1 Inohana, Chuo-ku, Chiba 260-8670, Japan

**Keywords:** oral squamous cell carcinoma, radioresistance, ICAM2, siRNA

## Abstract

We recently identified genes and molecular pathways related to radioresistance of oral squamous cell carcinoma (OSCC) using Affymetrix GeneChip. The current study focused on the association between one of the target genes, intercellular adhesion molecule 2 (*ICAM2*), and resistance to X-ray irradiation in OSCC cells, and evaluated the antitumor efficacy of combining ICAM2 small interfering RNA (siRNA) and X-ray irradiation. Downregulation of ICAM2 expression by siRNA enhanced radiosensitivity of OSCC cells with the increased apoptotic phenotype via phosphorylation (ser473) of AKT and activation of caspase-3. Moreover, overexpression of ICAM2 induced greater OSCC cell resistance to the X-ray irradiation with the radioresistance phenotype. These results suggested that ICAM2 silencing is closely related to sensitivity of OSCC cells to radiotherapy, and that ICAM2 may be an effective radiotherapeutic target for this disease.

Radiation therapy has played an important role in controlling tumour growth in many patients with cancer. In patients with oral squamous cell carcinoma (OSCC), radiation therapy is currently the standard adjuvant treatment. However, radiation therapy is sometimes ineffective as cancer cells can be resistant to radiation.

Our previous study showed that the radiosensitivity of OSCC cell lines differs greatly in their response to X-ray radiation, as assessed by clonogenic survival assay (Higo *et al*, 2005). In this study, we showed that in six OSCC cell lines HSC2 was the most radioresistant and HSC3 was the most radiosensitive cell line. Furthermore, we showed that 25 genes and their related molecular pathways related to cell growth and antiapoptosis were strongly associated with radiation resistance in OSCC (Ishigami *et al*, 2007). Of them, six were reported to be related to cell proliferation and antiapoptosis, that is, *ID1* (Nakajima *et al*, 1998; Zhang *et al*, 2004b), *ID3* (Plowright *et al*, 2000; Kowanetz *et al*, 2004), *FGFR3* (Gómez-Román *et al*, 2005), *PEG10* (Okabe *et al*, 2003; Tsou *et al*, 2003; Hu *et al*, 2004), intercellular adhesion molecule 2 (*ICAM2*) (Perez *et al*, 2002), and *MMP13* (McCawley and Matrisian, 2000; Chang and Werb, 2001; Corte *et al*, 2005), and we validated that the expression levels increased substantially in radioresistant cells compared with radiosensitive cells. Among the six genes identified, *ICAM2*, whose mRNA level in unirradiated HSC2 (radioresistant cells compared with unirradiated HSC3 (radiosensitive) cells was highest in our previous microarray results (Ishigami *et al*, 2007), underwent further functional analysis.

Intercellular adhesion molecule 2 has been thought to play a role in lymphocyte recirculation by blocking LFA-1-dependent cell adhesion and mediating adhesive interactions important for an antigen-specific immune response, NK cell-mediated clearance, lymphocyte recirculation, and other cellular interactions important for immune response and surveillance (Staunton *et al*, 1989; Li *et al*, 1995; Helander *et al*, 1996; Carpenito *et al*, 1997; Lehmann *et al*, 2003). However, ICAM2 has been reported to be a mediator for a survival signal sufficient to block apoptosis by activation of the PI3K/AKT pathway (Perez *et al*, 2002). In addition, another recent study has reported that absence of ICAM2 expression resulted in impaired angiogenesis *in vitro* and *in vivo*, and that ICAM2-deficient cells were defective in *in vitro* migration and increased apoptosis (Huang *et al*, 2005). It has been shown that *ICAM2*-expressing cells may be resistant to apoptosis induced by anticancer agents, including radiation. Therefore, we hypothesised that *ICAM2* inhibitors could enhance the effect of radiation on cancer cells that constitutively express *ICAM2*. Small-interfering RNA has been used widely to silence gene expression and has been evaluated as an attractive tool for use in therapeutics of many cancers (Gao *et al*, 2005; Zhang *et al*, 2005; Amarzguioui *et al*, 2006; Halder *et al*, 2006; Hosaka *et al*, 2006). So, we evaluated whether radiosensitivity of oral cancer cells is determined by cellular *ICAM2* by using siRNA targeted against *ICAM2* gene. Furthermore, we analysed the radiosensitivity of OSCC cells by upregulating *ICAM2* gene expression using expression vector encoding ICAM2 cDNA.

## MATERIALS AND METHODS

### Cell lines and culture conditions

The human OSCC-derived cell lines HSC2 and HSC3 (Human Science Research Resources Bank, Osaka, Japan) were prepared for this study. The cells were maintained in Dulbecco's modified Eagle's medium (DMEM) (Sigma Chemical Co., St Louis, MO, USA) supplemented with 10% heat-inactivated foetal bovine serum and 50 U ml^−1^ penicillin and streptomycin. All cultures were grown at 37°C under a humidified atmosphere of 5% carbon dioxide for routine growth.

### siRNA, transfection reagents, and transfection of siRNAs in HSC2 (radioresistant) cells

Small-interfering RNAs were obtained from Dharmacon Research Inc. (Lafayette, CO, USA). *SMART* pool siRNA targeting *ICAM2* consists of four siRNAs targeting multiple sites on *ICAM2* (*ICAM2*-siRNAs). The sequences for *ICAM2*-siRNAs are 5′-AAGCAGGAGUCAAUGAAUU-3′, 5′-UAACCAGCCUGAAGUGGGU-3′, 5′-UGAGAAGGUAUUCGAGGUA-3′, and 5′-ACGAACAGGCUCAGUGGAA-3′ (si*GENOME SMART*pool, M-11182-00-0005, Human *ICAM2*, NM_000873). Positive- and negative-control siRNAs were purchased from Dharmacon. Two negative controls were used in this study, that is, vehicle control (treated with only Dharma*FECT*1 reagent) and si*CONTROL* non-targeting siRNA pool (D-001210-01-05; non-targeting siRNA (siNT)). Cyclophilin *β* (siCONTROL Cyclophilin *β* siRNA, D-001136-01-05) was used as positive silencing control to ascertain transfection efficiency. Cells were transfected with siRNAs using Dharma*FECT*1 reagent (Dharmacon).

To confirm whether *ICAM2* gene is related to radioresistance, we performed an siRNA experiment to inhibit the expression of *ICAM2* in HSC2 (radioresistant) cell line that previously reported the expression of *ICAM2* as being higher than HSC3 (radiosensitive) cell line (Ishigami *et al*, 2007). HSC2 Cells were plated in antibiotic-free DMEM at a density of 200 000 cells 4 ml^−1^ in 60-mm dishes. After 24 h, the cells were transfected with 100 nmol l^−1^ siRNA in Dharma*FECT*1 reagent according to the manufacturer's instructions. Briefly, 8 *μ*l Dharma*FECT*1 was diluted in 392 *μ*l of serum-free medium and incubated at room temperature for 5 min. In a separate tube, 200 *μ*l of 2 *μ*mol l^−1^ siRNA was diluted in 200 *μ*l of serum-free medium at room temperature for 5 min. Diluted Dharma*FECT*1 (400 *μ*l) was added to the diluted siRNA and the complex was incubated for 20 min at room temperature. The cells were washed with antibiotic-free DMEM and 3.2 ml antibiotic-free DMEM was added to each dish. Small interfering RNA+Dharma*FECT*1 complex (800 *μ*l) was added gently to the dish. The final concentration of siRNA was 100 nmol l^−1^. Control cells were treated with the only medium (HSC2 control), the same amount of transfection reagents (vehicle control), the 100 nmol l^−1^ siNT (negative control), and the 100 nmol l^−1^ cyclophilin *β* siRNA (siCyclophilin *β*) (positive silencing control). After 4 h of transfection, the medium of cells treated with *ICAM2*-siRNAs (siRNA targeted to ICAM2 (siICAM2)) and control cells was replaced with fresh medium, and these were incubated at 37°C in 5% CO2 for 48 h to 120 h before performing experiments.

### Transient transfection of ICAM2 DNA

To verify whether *ICAM2* gene is related to radioresistance, we performed overexpression of *ICAM2* gene in HSC3 (radiosensitive) cell line that previously reported the expression of *ICAM2* as being higher than HSC2 (radioresistant) cell line (Ishigami *et al*, 2007). Human *ICAM2* cDNA was cloned into a pME18SFL3 expression vector (TOYOBO, Osaka, Japan) for transient transfection experiments. HSC3 cell lines were transfected with pME18SFL3 encoding *ICAM2* cDNA using the FuGENE HD transfection reagent (Roche Diagnostics GmbH, Mannheim, Germany). Mock transfection of HSC3 cell line cultures with the FuGENE HD transfection reagent alone was used as vehicle controls. Transfection efficiency was confirmed by real-time quantitative reverse transcriptase–polymerase chain reaction (qRT–PCR) and western blot analysis. These analyses were performed as described below.

### Irradiation using X-ray

The cells were irradiated with four single radiation doses (2, 4, 6, and 8 Gy) using X-ray irradiation equipment (MBR-1520R-3; Hitachi, Tokyo, Japan) operated at 150 V and 20 mA with AL filtration, at a dose of 2.1 Gy min^−1^.

### Isolation of RNA

Total RNA was extracted from X-ray-irradiated and unirradiated cells with TRIzor reagent (Invitrogen Life Technologies, Carlsbad, CA, USA) according to the manufacturer's instructions. The quality of the total RNA was determined using Bioanalyzer (Agilent Technologies, Palo Alto, CA, USA).

### Preparation of cDNA

Total RNA was extracted from cells using TRIzor reagent. Five micrograms of total RNA of each sample were reversed transcribed to cDNA using Ready-To-Go You-Prime First-Strand Beads (GE Healthcare, Little Chalfort, Buckinghamshire, UK) and oligo (dT) primer (Sigma Genosys, Ishikari, Japan), according to the manufacturers’ protocol.

### Analysis of mRNA expression by real-time qRT–PCR

Quantitative reverse transcriptase–polymerase chain reaction was performed to validate mRNA expression with a single method using a LightCycler FastStart DNA Master SYBR Green I kit (Roche Diagnostics GmbH), according to the procedure provided by the manufacturer. The oligonucleotides used as primers were 5′-GATCCAGGGCGGAGACTTC-3′ and 5′-GCCCGTAGTGCTTCAGTTTGA-3′ for *Cyclophilin β* mRNA, 5′-CATCTCTGCCCCCTCTGCTGA-3′ and 5′-GGATGACCTTGCCCACAGCCT-3′ for glyceraldehyde-3-phosphate dehydrogenase (*GAPDH*) mRNA, and 5′-ATTCAACAGCACGGCTGACA-3′ and 5′-CAGGCTCATAGATCTCCAACATCT- 3′ for *ICAM2* mRNA. Using LightCycler apparatus, we carried out PCR reactions in a final volume of 20 *μ*l of a reaction mixture consisting of 2 *μ*l of FastStart DNA Master SYBR Green I mix, 3 mM MgCl_2_, and 1 *μ*l of the primers, according to the manufacturer's instructions. Subsequently, the reaction mixture was loaded into glass capillary tubes and subjected to initial denaturation at 95°C for 10 min, followed by 33–45 rounds of amplification at 95°C (10 s) for denaturation, 62–68°C (10 s) for annealing, and 72°C for extension, with a temperature slope of 20°C s^−1^, performed with LightCycler. The transcript amount for the genes was estimated from the respective standard curves and normalised to the *GAPDH* transcript amount determined in the corresponding samples.

### Protein extraction

Protein was extracted from the cells, which were washed twice with phosphate-buffered saline, scraped into a tube with lysis buffer (7 M urea, 2 M thiourea, 4% w/v CHAPS, and 10 mM Tris, pH 8), and incubated at 4°C for 10 min. Cell extracts were lysed by sonication (3 × 10-s pulses on ice) and centrifuged at 13 000 **g** for 10 min at 4°C. The supernatant containing the cell proteins then was recovered. Protein concentration was determined using a commercial Bradford reagent (Bio-Rad, Richmond, CA, USA) and adjusted to 1 mg ml^−1^ with lysis buffer.

### Western blot analysis

Protein extracts (15 *μ*g) were electrophoresed on 12.5% sodium dodecyl sulfate-polyacrylamide gel electrophoresis gels, transferred to polyvinylidene difluoride (PVDF) membrane (Bio-Rad, Hercules, CA, USA), and blocked for 1 h at room temperature in 0.3% skimmed milk. Immunoblot PVDF membranes were washed with Tris-buffered saline Tween-20 (TBST: 10 mM Tris-HCl (pH 8.5), 150 mM NaCl, and 0.1% Tween-20) thrice and probed with 2 *μ*g ml^−1^ affinity-purified mouse anti-human ICAM2 monoclonal antibody (R&D Systems Inc., Minneapolis, MN, USA), 1 *μ*g ml^−1^ affinity-purified rabbit anti-human/mouse/rat specific AKT antibody (Rockland Inc., Gilbertsville, PA, USA), and 1 *μ*g ml^−1^ affinity-purified mouse anti-human p-AKT (pS437) antibody (BIOMOL International, L.P., Plymouth Meeting, PA, USA) overnight at room temperature. For cyclophilin *β* protein and *β*-actin protein, 1 *μ*g ml^−1^ affinity-purified rabbit anti-human cyclophilin *β* polyclonal antibody (Abcam Ltd, Cambridge, UK) and 1 *μ*g ml^−1^ affinity-purified mouse anti-human *β*-actin monoclonal antibody (Abcam Ltd) were used for 2 h at room temperature. Polyvinylidene difluoride membranes were washed again and incubated with 1 : 10 000 of Envision+(Dako Japan Inc., Kyoto, Japan) as secondary antibody coupled to horseradish peroxidase-conjugated anti-mouse or ant-rabbit IgG antibody for 2 h at room temperature. Finally, membranes were incubated using the ECL Plus Western Blotting Detection System (GE Healthcare, Carpinteria, Buckinghamshire, UK) and immunoblotting result was visualised by exposing the membrane to Fuji Medica X-Ray film RX-U (FUJIFILM, Kanagawa, Japan). Signal intensities were quantitated using ImageJ software (1.38x version)(NIH, Bethesda, MD, USA).

### Cell-proliferation assay

To determine the effect of *ICAM2* siRNA on cell proliferation, HSC2 cells transfected with non-targeting or *ICAM2* siRNA (100 nmol l^−1^) were seeded in 12-well plates at a density of 1 × 10^4^ viable cells per well. Mock-transfected cells were treated with Dharma*FECT*1 reagent as vehicle controls but not siRNA. At the indicated time point, cells were trypsinised and counted using a haemocytometer in triplicate samples. The effect on cell proliferation was investigated in the same manner as HSC3 cells treated with expression vector of *ICAM2* DNA.

### Clonogenic cell survival assay

HSC2 cells were transfected as above with the vehicle, siNT, and siICAM2. At 72, 84, and 96 h after transfection, the cells were trypsinised, counted, and the appropriate number of cells were plated in 60-mm dishes and allowed to attach for 24 h. After 24 h, the cells were irradiated (2, 4, 6, 8 Gy) and incubated for 8–10 days. The colonies were stained with crystal violet (Sigma Chemical Co.), and colonies of 50 cells or greater were counted. Clonogenic fractions of irradiated cells were normalised to the plating efficiency of unirradiated controls. Likewise, colonies of HSC3 cells transfected with expression vector of *ICAM2* DNA were measured.

### *In situ* cell apoptosis detection assay

HSC2 cells were plated in 24-well dishes at a density of 2 × 10^4^ cells per well. After 24 h, HSC2 cells were treated with siRNA (vehicle, siNT, siICAM2). After incubation for 120 h, cells were analysed for apoptosis by terminal deoxynucleotidyl transferase (TdT)-mediated dUTP nick-end labelling (TUNEL) procedure, using an *in situ* apoptosis detection kit (Takara Bio, Shiga, Japan). Briefly, cells were fixed in 4% paraformaldehyde for 15 min. After treatment with permeabilisation buffer (Takara Bio) for 2 min on ice, the cells were incubated with TdT enzymes plus labeling safe buffer (Takara Bio) at 37°C. After 1.5 h, the cells were washed with phosphate-buffered saline and observed by fluorescence microscopy. Cells undergoing apoptosis were specifically labelled with fluorescein-dUTP with high sensitivity, allowing immediate detection by fluorescence microscopy. Apoptotic cells were counted and expressed as a percentage of the total cells. The experiments were performed in triplicate.

### Caspase-3 activity assay

We performed three different assays for caspase-3 activity. Two assays (using caspase-3 detection kit; Calbiochem, Oxford, UK) measured the amount of activated caspase-3 *in situ* in living cells by a caspase-3 inhibitor (DEVD-FMK) conjugated to FITC as a fluorescent *in situ* marker. Fluorescein isothiocyanate-DEVD-FMK is cell-permeable and nontoxic and irreversibly binds to activated caspase-3 in apoptotic cells. The FITC label allows direct detection of activated caspase-3 by fluorescence microscopy or a fluorescence microplate reader. In our initial experiment, FITC fluorescence was conjugated to the inhibitor, thereby registering a fluorescent label detected by fluorescence microscopy in cells in which caspase-3 had been activated. Both cells treated with and without ICAM2 siRNA (control, vehicle, and siNT) were compared. Caspase-3 active cells were counted and expressed as a percentage of the total number of cells. The experiments were performed in triplicate. For the next experiment, we used a microplate reader to study the effect. Data were expressed as the difference in activity among the four data points (control, vehicle, siNT, and siICAM2) and indicated the amount of caspase-3 activity that remained. The kit was used according to the manufacturer's instructions. Briefly, cells were treated with or without ICAM2 siRNA for 120 h, after which they (about 1 × 10^6^ ml^−1^) were incubated for 1 h with FITC-DEVD-FMK at 37°C. The cells then were washed twice by washing buffer and then the fluorescence intensity was measured by fluorescence microscopy and using a fluorescence microplate reader (Wallac 1420 ARVOsx Multilabel Counter; Perkin-Elmer, Chiba, Japan), with excitation at approximately 485 nm and emission detection at approximately 535 nm. Finally, we performed activated caspase-3 western blot analysis. Monoclonal antibody to active caspase-3 (Genlantis Inc., San Diego, CA, USA) was used for detection. Western blot analysis for activated caspase-3 was performed as described previously.

## RESULTS

### Transfection efficiency of siRNAs

**Figure 1:**
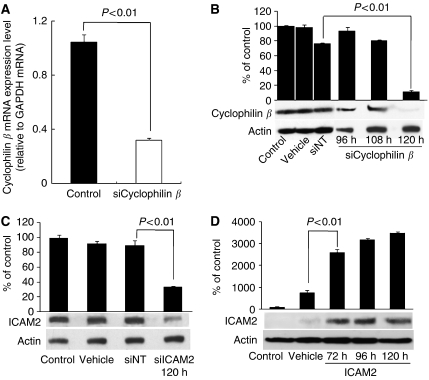
Effect of siRNAs. (**A**) Expression of cyclophilin *β* mRNA in non-transfected HSC2 cells (control) and HSC2 cells transfected with siCyclophilin *β* (*P*<0.01, Student's *t*-test). (**B**) Western blot analysis of cyclophilin *β* protein (positive control) in HSC2. The cells were transfected with 100 nmol l^−1^ siNT and siCyclophilin *β* and analysed at 96, 108, and 120 h. Cyclophilin *β* and actin bands were scanned and quantitated as described under Materials and Methods. The values obtained from densitometric analysis of each cyclophilin *β* protein first were normalised to actin protein levels and then expressed as the percentage of the values of control. Cyclophilin *β* proteins were significantly inhibited (*P*<0.01, Student's *t*-test) in cells transfected with siCyclophilin *β* at 120 h. There is no change in cyclophilin *β* in cells transfected with vehicle and siNT negative controls siRNA. (**C**) Western blot analysis of ICAM2 protein in HSC2 cells transfected with vehicle, siNT, and siICAM2. The cells were transfected with 100 nmol l^−1^ siRNAs and analysed at 120 h. Intercellular adhesion molecule 2 and actin bands were scanned and quantitated as described under Materials and Methods. The values obtained from densitometric analysis of each ICAM2 protein first were normalised to actin protein levels and then expressed as the percentage of the values of control. The ICAM2 proteins were significantly inhibited (*P*<0.01, Student's *t*-test) in cells transfected with siICAM2 at 120 h. (**D**) Western blot analysis of ICAM2 in ICAM2-overexpressing HSC3 cells. The cells were examined 72, 96, and 120 h after transient transfection of expression vector encoding ICAM2 cDNA. Actin was used as a loading control. Intercellular adhesion molecule 2 and actin bands were scanned and quantitated as described under Materials and Methods. The values obtained from densitometric analysis of each ICAM2 protein first were normalised to actin protein levels and then expressed as the percentage of the values of control. The ICAM2 protein levels increased significantly (*P*<0.01, Student's *t*-test) in cells transfected with the expression vector of ICAM2 DNA at 72, 96, and 120 h.

To ascertain whether conditions for RNA inhibition were optimal and that transfection efficiency was satisfactory, siCyclophilin *β* was used as a positive silencing control. In real-time qRT–PCR analysis, *cyclophilin β* mRNA expression decreased by 69% with siCyclophilin *β* 48 h after transfection (Figure 1A). In western blot analysis, cyclophilin *β* was detected as a single band. In cells transfected with siCyclophilin *β*, the band diminished significantly (*P*<0.01, Student's *t*-test), confirming high transfection efficiency 120 h after transfection compared with the vehicle, si*CONTROL* siNT, and the non-transfected control cells. HSC2 cells transfected with siCyclophilin *β* showed little change in the band of cyclophilin *β* 96 and 108 h after transfection compared with controls (Figure 1B).

### ICAM2 protein levels in HSC2 cells transfected with siICAM2 and control siRNAs

Figure 1C shows western blot analysis of ICAM2 protein expression in HSC2 cells 120 h after transfection with siRNAs. This time point was selected by a positive silencing control test in an siRNA transfection experiment (Figure 1B). The ICAM2 protein levels in cells transfected with vehicle, siNT, and siICAM2-transfected cells were comparable to those in non-transfected cells. In cells transfected with 100 nmol l^−1^ siICAM2, ICAM2 protein level decreased significantly (*P*<0.01, Student's *t*-test) compared with non-transfected, vehicle-transfected, and the siNT-transfected cells (Figure 1C). In addition, qRT–PCR analysis showed that *ICAM2* mRNA expression decreased by 67, 97, and 96% in HSC2 cells with siICAM2 compared with non-transfected cells at 24, 48, and 72 h, respectively, after transfection.

### ICAM2 protein levels in HSC3 cells transfected with ICAM2 DNA

Figure 1D shows western blot analysis of ICAM2 protein expression in HSC3 cells 72, 96, and 120 h after transfection with expression vector encoding *ICAM2* cDNA. The ICAM2 protein level increased significantly (*P*<0.01, Student's *t*-test) in cells transfected with *ICAM2* DNA compared with vehicle and non-transfected control cells (Figure 1D). Furthermore, qRT–PCR analysis showed that the level of *ICAM2* mRNA expression increased significantly (*P*<0.01, Student's *t*-test) by 4.5 × 10^6^ and 9.4 × 10^6^-fold in HSC3 cells with expression vector encoding ICAM2 cDNA compared with non-transfected cells 48 and 72 h, respectively, after transfection.

### Effect of ICAM2 siRNA and expression vector encoding ICAM2 cDNA on cell growth

**Figure 2:**
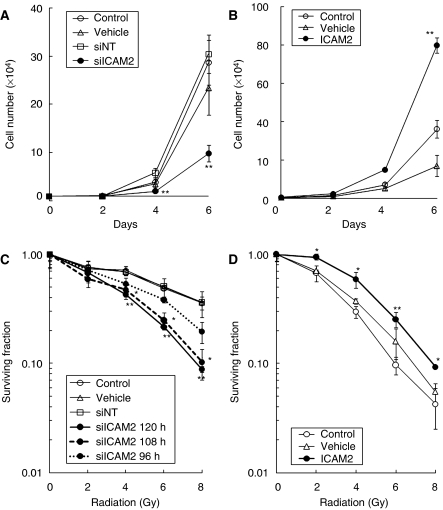
Effect of *ICAM2* expression on cell growth and cell survival. (**A**) HSC2 cells were transfected with 100 nmol l^−1^ ICAM2 siRNA (siICAM2), siNT, or vehicle. At the indicated time points, the cells were trypsinised and counted using a haemocytometer in triplicate samples. The results represent the mean±s.d. The asterisks indicate significant differences between siNT and ICAM2 siRNA (^**^P<0.01, Student's *t*-test). (**B**) HSC3 cells were transfected with expression vector encoding *ICAM2* cDNA or vehicle. At the indicated time points, the cells were trypsinised and counted using a haemocytometer in triplicate samples. The results are expressed as the mean±s.d. The asterisks indicate significant differences between the vehicle and ICAM2 transfectant (^**^P<0.01, Student's *t*-test). (**C**) The effect of radiation on the clonogenic survival of HSC2 cells transfected with the vehicle, siNT, and siICAM2. At 72, 84, and 96 h after transfection; the cells were trypsinised and plated for clonogenic survival assay for siICAM2 at 96 h, siICAM2 at 108 h, and siICAM2 at 120 h, respectively. After 24 h, when the cells had attached, they were irradiated. Colonies were stained with crystal violet and counted after 8–10 days. Each experiment was repeated at least three times; the error bars represent ±s.d. The significance of the difference between siNT (open square) and siICAM2 (closed circle) is indicated by asterisks (^**^P<0.01, ^*^P<0.05, Student's *t*-test). (**D**) The effect of radiation on the clonogenic survival of HSC3 cells overexpressing ICAM2, vehicle treated cells, and untreated control. The clonogenic survival of HSC3 cells was calculated in the same way as for HSC2 cells. The significance of the difference between the vehicle (open triangles) and ICAM2-overexpressing cells (closed circles) is indicated by asterisks (^**^P<0.01, ^*^P<0.05, Student's *t*-test).

To determine the effect of *ICAM2* siRNA and expression vector encoding *ICAM2* cDNA on the growth of the cancer cell lines, a series of cell growth experiments was carried out. The growth curves of HSC2 cells showed that treatment with ICAM2 siRNA inhibited cell growth over a period of 6 days, but cell growth was not inhibited by vehicle and siNT (Figure 2A). Furthermore, the growth curve of HSC3 cells showed that treatment with expression vector encoding *ICAM2* cDNA increased cell growth over a period of 6 days compared with the vehicle (Figure 2B).

### Radiosensitivity of HSC2 cells transfected with siICAM2

The plating efficiencies of unirradiated HSC2 cells (mean±s.d., *n*=3) were 0.278±0.038 (control), 0.270±0.032 (vehicle), 0.262±0.031 (siNT), 0.258±0.031 (siICAM2, 120 h), 0.275±0.066 (siICAM2, 108 h), and 0.220±0.057 (siICAM2, 96 h). The difference in the plating efficiency of siICAM2 cells compared with vehicle or siNT cells was not significant. Figure 2C shows the radiation survival curves of untreated control cells and cells transfected with siRNAs. These data were used to calculate dose D_37_ that is required to reduce survival to 37% (Toulany *et al*, 2006). For HSC2 cells, dose D_37_ values for control, vehicle, siNT, siICAM2 at 108 h, siICAM2 at 96 h, and siICAM2 at 120 h were 7.6, 7.6, 7.7, 6.0, 4.7, and 4.4 Gy, respectively. Compared with the vehicle-treated control, at the 37% survival level, the radiosensitivity of cells transfected with siICAM2 at 120 h was enhanced by a dose-modifying factor of 1.73 and the radiosensitivity of cells transfected with siNT was 0.99-fold.

### Cells transfected with siICAM2 at 120 h compared with cells transfected with siNT

The radiosensitivity of cells transfected with siICAM2 at 120 h was enhanced by a dose-modifying factor of 1.75 for siNT. Moreover, survival of HSC2 cells transfected with siICAM2 at 120 h decreased significantly (*P*<0.01, Student's *t*-test) after 4, 6, and 8 Gy of radiation compared with that of HSC2 cells treated with siNT (Figure 2C). This indicates that radiation-induced sublethal damage is severe when *ICAM2* expression is inhibited.

### Radiosensitivity of HSC3 cells transfected with expression vector of ICAM2 DNA

In HSC3 cells, the plating efficiencies at 0 Gy (mean±s.d., *n*=3) were 0.211±0.030 (control), 0.262±0.003 (vehicle), and 0.232±0.030 (ICAM2). The difference in the plating efficiency of cells transfected with expression vector of *ICAM2* DNA, compared with vehicle or untreated control cells, was not significant. The radiation survival curves of cells are shown in Figure 2D. In cells transfected with expression vector encoding ICAM2 cDNA, compared with cells transfected with vehicle and untreated control cells, the radiosensitivity of the transfectant decreased. For HSC3 cells, dose D_37_ values for control, vehicle, and the transfectant were 3.4, 4, and 5 Gy, respectively. The resulting dose-modifying factors were 0.80 for the vehicle and 0.68 for the untreated control cells at the 37% survival level. Furthermore, the rate of cell survival of the transfectant increased significantly (^**^P<0.01, ^*^P<0.05, Student's *t*-test) after 2, 4, 6, and 8 Gy of radiation, compared with that of cells treated with vehicle (Figure 2D).

### Correlation of ICAM2 expression and apoptosis

**Figure 3:**
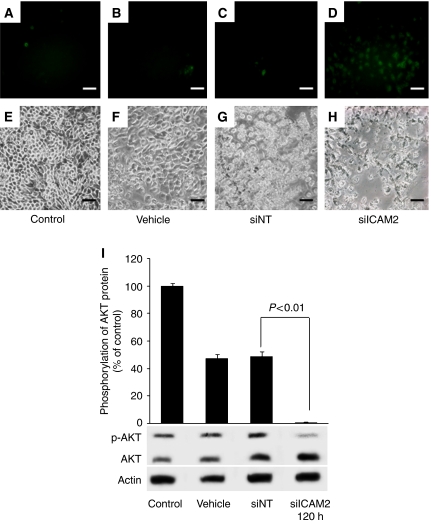
The effects of *ICAM2* inhibition on apoptosis in HSC2 cells. DNA fragmentation of apoptotic cells is detected by TUNEL assay. (**D**) HSC2 cells were treated with 100 nmol l^−1^ siICAM2 for 120 h. Many apoptotic cells are detected in HSC2 cells with siICAM2. (**A**–**C**) Few apoptotic cells are detected in cells transfected with the vehicle, siNT, and in untreated control cells. (**E**–**H**) The lower panels are phase-contrast micrographs showing total cells. Bar=35 *μ*m. (**I**) Western blot analyses of AKT and p-AKT (AKT phosphorylation at the Ser473 site) protein in HSC2 cells transfected with the vehicle, siNT, siICAM2, and in untreated control. The cells were transfected with 100 nmol l^−1^ siRNAs and analysed at 120 h. p-AKT and AKT bands were scanned and quantitated as described under Materials and Methods. The values obtained from densitometric analysis for each AKT proteins were first normalised to AKT protein levels and then expressed as the percentage of the values for the control. The p-AKT proteins were significantly inhibited (*P*<0.01, Student's *t*-test) in cells transfected with siICAM2 at 120 h.

To examine whether expression of *ICAM2* is the result of apoptosis, we analysed DNA fragmentation by TUNEL assay. As shown in Figure 3A–D, positive TUNEL labelling in apoptotic cells was bright. Figure 3D clearly shows that apoptosis was induced in some cells but not in others (Figure 3A–C). The percentages of apoptotic cells in HSC2 cells transfected with the vehicle, siNT, siICAM2, and in the untreated control cells were 1.40±0.09, 2.56±0.29, 26.4±8.81, and 1.05±0.14%, respectively. Data are expressed as the mean±s.d. of three independent experiments. The difference between siNT and siICAM2 reached significance (*P*<0.01, Student's *t*-test).

### ICAM2-induced AKT phosphorylation

Recent studies have reported that ICAM2 activated the protein kinase B (PKB)/AKT pathway leading to inhibition of apoptosis (Perez *et al*, 2002). In this pathway, phosphorylation (Ser473) of AKT protein induced antiapoptosis. To investigate the correlation of ICAM2 and AKT phosphorylation, we performed western blot analysis with antibody recognising AKT and AKT phosphorylation at Ser473 site (Figure 3I). Figure 3I shows that HSC2 cells treated with siICAM2 resulted in lower AKT phosphorylation level than cells treated with the vehicle, siNT, and the untreated control cells.

### Caspase-3 activity assay

**Figure 4:**
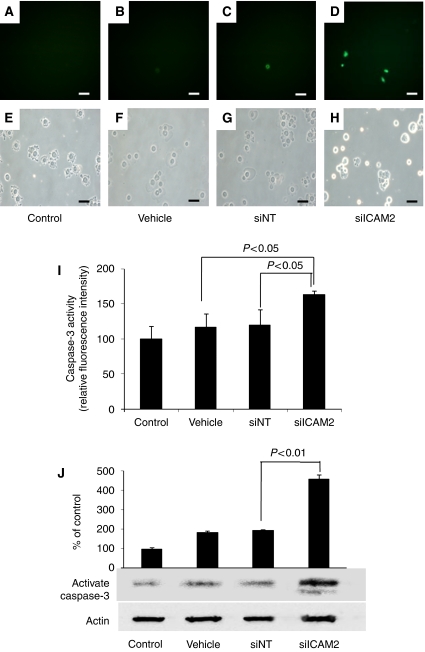
The effect of *ICAM2* inhibition on caspase-3 activity in HSC2 cells. (**D**) HSC2 cells treated with 100 nmol l^−1^ siICAM2 for 120 h. Relatively more caspase-3 active cells are detected in HSC2 cells with siICAM2. (**A**–**C**) Few caspase-3 active cells are detected in cells transfected with the vehicle, siNT, and in untreated control cells. (**E**–**H**) The lower panels are phase-contrast micrographs showing total cells. Bar=35 *μ*m. (**I**) HSC2 cells were treated with ICAM2 siRNA for 120 h and the activity of caspase-3 was measured using the caspase-3 detection kit with a microplate reader as described under Materials and Methods. The results are expressed as mean±s.d. of three independent experiments. Caspase-3 activity is increased significantly (*P*<0.05, Student's *t*-test) in cells transfected with siICAM2 at 120 h. (**J**) Western blot analysis of active caspase-3 protein in HSC2 cells transfected with the vehicle, siNT, and siICAM2. Cells were transfected with 100 nmol l^−1^ siRNAs and analysed at 120 h. Active caspase-3 and actin bands were scanned and quantitated as described under Materials and Methods. The values obtained from densitometric analysis for each active caspase-3 protein were first normalised to actin protein levels and expressed as the percentage of the control values. Active caspase-3 protein levels are increased significantly (*P*<0.01, Student's *t*-test) in cells transfected with siICAM2 at 120 h.

We investigated the role of caspase-3 in this process to determine the mechanism by which apoptosis occurred. Figure 4A–D show the green FITC signal present within the siICAM2 cells; control, vehicle, and siNT cells displayed a small activated caspase-3 signal. The percentages of caspase-3 active cells in HSC2 cells transfected with the vehicle, siNT, siICAM2, and the untreated control cells were 2.60±2.30%, 4.00±0.61%, 20.7±0.71%, and 1.45±2.51%, respectively. Data are expressed as the mean±s.d. of three independent experiments. The difference between siNT and siICAM2 reached significance (*P*<0.01, Student's *t*-test). The second assay (caspase-3 activity assay) (Figure 4I) showed that caspase-3 activity was significantly elevated in cells with ICAM2 siRNA for 120 h compared with cells with vehicle and siNT (*P*<0.05, Student's *t*-test). Quantification of relative fluorescence intensity revealed that siICAM2 increased caspase-3 enzymatic activity 1.36-fold compared with siNT. The third study examined the protein levels of active caspase-3. Figure 4J shows that HSC2 cells treated with siICAM2 had significantly higher (*P*<0.01, Student's *t*-test) active caspase-3 protein levels than cells treated with the vehicle, siNT, and the untreated control cells.

## DISCUSSION

*ICAM2*, whose mRNA level was upregulated in radioresistant OSCC cells in our previous study (Ishigami *et al*, 2007), was one of candidate genes selected as radioresistant genes of OSCC cells by microarray analysis using Affymetrix GeneChip.

The current study was designed to examine whether ICAM2 is functionally associated with radiosensitivity of OSCC *in vitro*. In the previous report, little has been mentioned about the role of ICAMs in regard to radioresistance. Intercellular adhesion molecule 1 expression was shown to be elevated by hypoxia and radiation (Zünd *et al*, 1997; Meineke *et al*, 2002). Intercellular adhesion molecule 3 expression was reported to associate with radioresistance in cervical cancer (Chung *et al*, 2005).

In our study, the results indicated that ICAM2 inhibition induces radiosensitive *in vitro* (Figure 2C). A further important point is that radiosensitisation of cells may depend on the extent of ICAM2 protein inhibition. Because HSC2 cells were incubated for a longer time with siICAM2, they became more radiosensitive (Figure 2C), suggesting that radiosensitisation of HSC2 cells may be determined by the amount of ICAM2 protein present in the cells at the time of irradiation. Moreover, ICAM2 overexpression induces radioresistance *in vitro* (Figure 2D). These results may indicate that regulation of ICAM2 protein is correlated with radiosensitivity. Further, Figure 2A and B show that regulation of ICAM2 protein is related to cell growth. The question that needs to be answered is what mechanisms play a role in the relation between the regulation of the ICAM2 protein and these results.

Intercellular adhesion molecule 2-induced activation of AKT kinase resulted in the activation of several downstream effectors as detected by phosphorylation of BAD, GSK3, FKHR, and AFX, all of which can contribute to cell survival (Zha *et al*, 1996; Brunet *et al*, 1999). The PI3/AKT-signalling system is a general mediator of extracellular stimuli, including growth factors, cytokines, and adhesion, to extracellular matrices (Downward, 1998). Phosphatidylinositol-3,4,5-triphosphate binds to the pleckstrin domain of AKT and recruits AKT to the membrane, where it becomes dually phosphorylated (Alessi *et al*, 1996) and is then activated and phosphorylates a number of downstream effectors that contribute to cell survival. These studies indicated that ICAM2 activation might lead to an antiapoptic signal in a variety of cells. A recent study identified a pathway of ICAM2 activating the PI3K/AKT leading to inhibition of apoptosis (Perez *et al*, 2002). This pathway showed that ICAM2 induced tyrosine phosphorylation of ezrin and PI3K kinase membrane translocation, resulting in phosphatidylinositol-3,4,5 production, PDK-1 and AKT activation, and subsequent phosphorylation of AKT targets BAD, GSK3, and FKHR. The previous studies may have presumed that the differences in cell growth and radiosensitivity are caused by mechanisms of antiapoptotic effect induced by ICAM2. Consequently, we postulated that ICAM2 induced antiapoptosis in OSCCs as well as the mechanism of the PKB/AKT pathway activation previously reported (Perez *et al*, 2002). In addition, AKT phosphorylation at Ser473 was suggested to be correlated perfectly with antiapoptosis in lymphocytes (Perez *et al*, 2002). Therefore, we examined AKT phosphorylation (Ser473) and cell apoptosis to confirm the relation between ICAM2 and antiapoptosis via the PKB/AKT pathway in OSCC cells. The results of our experiment showed that cells with siICAM2 induced a decrease in AKT phosphorylation (Ser473) (Figure 3I). To detect cell apoptosis, we performed caspase-3 activity assays and TUNEL assay. We evaluated caspase-3 activation in cells treated with ICAM2 siRNA. HSC2 cells treated with siICAM2 showed increased caspase-3 activity compared with untreated or control cells (Figure 4). In the previous studies, the apoptotic pathway induced by decrease in p-AKT caused activation of caspase-3 (Ivins Zito *et al*, 2004; Zhang *et al*, 2004a; Zhuang *et al*, 2007). We considered that decreased p-AKT and increased activated caspase-3 induced apoptosis in cells treated with ICAM2 siRNA. Furthermore, results of the TUNEL assay showed increased apoptosis (Figure 3D). Accordingly, it is reasonable that inhibiting ICAM2 may contribute to radiosensitisation of OSCC cells by increased apoptosis via phosphorylation (Ser473) of AKT and activation of caspase-3.

Our study was similar to a previous study (Chung *et al*, 2005) in which the investigators used the same methods, that is, microarray analysis, siRNA, overexpression, and apoptosis assay. These two studies yielded similar results in that they both confirmed a relation between a gene and radioresistance. However, due to availability of clinical samples, our study differed from the other in that we could not analyse clinical specimens from patients. Our study, therefore, may be insufficient *in vivo*. Our study could not prove a relation between ICAM2 and radioresistance in OSCC cells *in vitro*.

In conclusion, *ICAM2* expression may be associated with radioresistance in OSCC cells, *ICAM2* siRNA may enhance the radiosensitivity of oral cancer cells, and ICAM2 may be an effective radiotherapeutic target of oral cancer and a marker for radiation sensitivity based on *in vitro* studies with microarray analysis.
